# Functional characterization of quorum sensing LuxR-type transcriptional regulator, EasR in *Enterobacter asburiae* strain L1

**DOI:** 10.7717/peerj.10068

**Published:** 2020-10-21

**Authors:** Yin Yin Lau, Kah Yan How, Wai-Fong Yin, Kok-Gan Chan

**Affiliations:** 1 International Genome Centre, Jiangsu University, Zhenjiang, China; 2 Division of Genetics and Molecular Biology, Institute of Biological Sciences, Faculty of Science, University of Malaya, Malaysia

**Keywords:** Transcriptional regulator, *Enterobacter asburiae*, β-galactosidase assays, Quorum sensing, *N*-acyl homoserine lactone

## Abstract

Over the past decades, *Enterobacter* spp. have been identified as challenging and important pathogens. The emergence of multidrug-resistant Enterobacteria especially those that produce *Klebsiella pneumoniae* carbapenemase has been a very worrying health crisis. Although efforts have been made to unravel the complex mechanisms that contribute to the pathogenicity of different *Enterobacter* spp., there is very little information associated with AHL-type QS mechanism in *Enterobacter* spp. Signaling via *N*-acyl homoserine lactone (AHL) is the most common quorum sensing (QS) mechanism utilized by Proteobacteria. A typical AHL-based QS system involves two key players: a *luxI* gene homolog to synthesize AHLs and a *luxR* gene homolog, an AHL-dependent transcriptional regulator. These signaling molecules enable inter-species and intra-species interaction in response to external stimuli according to population density. In our recent study, we reported the genome of AHL-producing bacterium, *Enterobacter asburiae* strain L1. Whole genome sequencing and in silico analysis revealed the presence of a pair of *luxI/R* genes responsible for AHL-type QS, designated as *easI/R*, in strain L1. In a QS system, a LuxR transcriptional protein detects and responds to the concentration of a specific AHL controlling gene expression. In *E. asburiae* strain L1, EasR protein binds to its cognate AHLs, *N*-butanoyl homoserine lactone (C4-HSL) and *N*–hexanoyl homoserine lactone (C6-HSL), modulating the expression of targeted genes. In this current work, we have cloned the 693 bp *luxR* homolog of strain L1 for further characterization. The functionality and specificity of EasR protein in response to different AHL signaling molecules to activate gene transcription were tested and validated with β-galactosidase assays. Higher β-galactosidase activities were detected for cells harboring EasR, indicating EasR is a functional transcriptional regulator. This is the first report documenting the cloning and characterization of transcriptional regulator, *luxR* homolog of *E. asburiae*.

## Introduction

Quorum sensing (QS) is a cell-to-cell communication system which is widely-used by bacteria as their network to monitor and regulate targeted gene expression as a function of cell density in different environment (Steindler et al., 2008). Signaling via *N*-acyl homoserine lactone (AHL) is a type of QS mechanism that is widely-utilized by most Proteobacteria (Miller & Bassler, 2001; Schaefer et al., 2008). To date, AHL is the most well-studied and characterized QS signaling molecule (Miller & Bassler, 2001). A typical AHL-based QS system involves two key players: a *luxI* gene homolog to synthesize AHLs and a *luxR* gene homolog, an AHL-dependent transcriptional regulator (Rutherford & Bassler, 2012).

The LuxR transcriptional regulator plays important roles to detect and respond to the concentration of the specific AHLs, thereby controlling gene expression of QS-regulated genes (Fuqua, 2006). Various studies have revealed that LuxR helps to coordinate the expression of a plethora of genes such as those involved in bioluminescence, biofilm formation, antibiotics biosynthesis as well as the production of pathogenetic and virulence factors (How et al., 2015; Parsek & Greenberg, 2000; Pesci et al., 1999; Rutherford & Bassler, 2012). LuxR is a member of the FixJ-NarL superfamily (Kahn & Ditta, 1991). It consists of two principal conserved domains: an N-terminal AHL-binding domain and a C-terminal DNA binding helix-turn-helix (HTH) domain (Choi & Greenberg, 1991; Hanzelka & Greenberg, 1995; Santos et al., 2012). In the absence of the cognate AHL, the N-terminal domain can fold over the HTH of the C-terminal domain, thus, blocking binding with the targeted promoter DNA. On the other hand, when the AHL concentration has reached its threshold level, the cognate AHL binds to the N-terminal “signal-binding” domain, resulting in LuxR conformation changes and promotes multimerization (Castang et al., 2006; Colton, Stabb & Hagen, 2015; Patankar & Gonzalez, 2009).

LuxR then binds to a specific DNA binding site called a *lux* box, which normally constitutes 20 nucleotides and located at 42.5 nucleotides upstream of the transcriptional start site (Devine, Shadel & Baldwin, 1989; Urbanowski, Lostroh & Greenberg, 2004). The presence of the imperfect dyad symmetry in the *lux* box sequence suggests that the DNA binding domains are multimeric and have a corresponding two-fold rotational symmetry (Antunes et al., 2008). Activation of the *lux* box leads to a remarkable increase in the level of AHLs and creates a positive-feedback loop.

LuxR proteins usually bind to AHLs produced by cognate LuxI synthases with both high specificity and high affinity (Gray et al., 1994). However, some studies revealed that LuxR homologs could detect up to seven related AHLs, but with a lower sensitivity (Steindler & Venturi, 2007). There is no distinct evidence that shows which residues determine AHL specificity, in terms of length and composition (Collins, Arnold & Leadbetter, 2005). Alteration in the acyl side chains and/or any replacement within the homoserine lactone ring can result in constitutive LuxR activity (Choi & Greenberg, 1991). Analogs of AHLs can have an inhibitory effect on LuxR dependent transcriptional activation by preventing the cognate AHL from binding with LuxI (Schaefer et al., 1996; Zhu et al., 2002).

Over the past decades, *Enterobacter* spp. have been identified as challenging and important pathogens (Sanders & Sanders, 1997). The emergence of multidrug-resistant Enterobacteria especially those that produce *Klebsiella pneumoniae* carbapenemase (Kitchel et al., 2009; Nordmann, Naas & Poirel, 2011; Tzouvelekis et al., 2012) have been a very worrying health crisis. Although efforts have been made to unravel the complex mechanisms that contribute to the pathogenicity of different *Enterobacter* spp., there is very little information associated with AHL-type QS mechanisms in *Enterobacter* spp. (Lau et al., 2018).

*Enterobacter asburiae* is a gram-negative bacillus that belongs to the *Enterobacter* genus, classified as the Enteric group 17 by Brenner et al., 1986. *E. asburiae* has been isolated from soil, water and a variety of human sources including urine, respiratory tracts, stools, wounds, and blood (Bi, Rice & Preston, 2009; Brenner et al., 1986; Koth et al., 2012; Paterson et al., 2005). This microorganism has also been found from a wide variety of crops such as rice, cucumber, and cotton (Asis & Adachi, 2004; Elbeltagy et al., 2001; McInroy & Kloepper, 1995). Previous works have identified some of the *E. asburiae* isolates as human pathogens while most of the strains were opportunistic pathogens that could cause different human diseases such as wound infection, community-acquired pneumonia, and soft tissue infections (Brenner et al., 1986; Cha et al., 2013; Koth et al., 2012; Stewart & Quirk, 2001). *E. asburiae* has been reported to produce the Bush group 1 β-lactamase enzyme constitutively at high levels which causes this bacterium to be resistant to most β-lactam antibiotics (Pitout et al., 1997). In recent years, cases of colistin-resistant *E. asburiae* have been reported (Kadar et al., 2015; Stewart & Quirk, 2001). Noteworthy, due to the increasing circulation of the *E. asburiae* especially in the nosocomial setting, *E. asburiae* has been identified as one of the emergent pathogens causing severe infections (De Florio et al., 2018).

As seen in recent years, whole-genome sequence (WGS) analysis is a common and popular platform for bacterial genomic studies (De Bona et al., 2008). WGS and in silico analysis enable us to gain more knowledge of virulence, antibiotic production, bioluminescence, biofilm formation, and the different pathogenic effects of the isolates in different environments or models of infection (Liu et al., 2016). Recently, a novel AHL-producing *E. asburiae* strain L1 has been isolated from lettuce leaves and its genome was completely sequenced. A previous study by Zhu et al. (2017) revealed that *E. asburiae* strain L1 exhibited the highest 16S rRNA sequence similarity with *E. asburiae* strain ATCC 35953 (type strain), which is a multidrug-resistant pathogen that was isolated from a human source (Brenner et al., 1986). This triggered our interest to further characterize *E. asburiae* strain L1 which was found to secrete *N*-butanoyl homoserine lactone (C4-HSL) and *N*–hexanoyl homoserine lactone (C6-HSL) (Lau et al., 2013). Analysis of the annotated genome led us to the discovery of a pair of *luxI/R* gene homologs (Lau, Yin & Chan, 2014). As LuxR is a key player in the AHL-based QS system, this has prompted us to further characterize the regulatory roles of the transcriptional protein on the expression of virulence and unknown genetic traits of strain L1. In this study, the *luxR* homolog, *easR*, was cloned and the response of the expressed protein, EasR, on different variants of AHLs was investigated using β-galactosidase assay.

## Materials and Methods

### Bacterial strains and growth conditions

All bacterial strains and plasmids used in this study are listed in Table S1. Strain L1 was grown in Luria-Bertani (LB) broth (1.0% w/v peptone, 0.5% w/v yeast extract, 1.0% w/v NaCl) or agar (Merck, Kenilworth, NJ, USA) at 37 °C (Lau et al., 2013). *Escherichia coli* strains were grown aerobically on LB media at 37 °C (for cells that harbored pGEM^®^-T and pMULTIAHLPROM recombinant plasmids) or at 30 °C (for cells that harbored pLNBAD recombinant plasmids). Liquid cultures were grown in an orbital shaking incubator at 250 rpm. When indicated, the transformed cells were grown in LB media supplemented with antibiotics in the following concentrations: 100 µg/ml ampicillin (Sigma, St. Louis, MO, USA), 20 µg/ml chloramphenicol or 10 µg/ml tetracycline (Sigma, St. Louis, MO, USA).

### Nucleotide sequence and bioinformatics analysis

The whole genome of strain L1 has been sequenced and annotated (Lau, Yin & Chan, 2014). Gene annotation and prediction of strain L1 LuxR-type transcriptional regulator was performed using SEED-based automated annotation system provided by the Rapid Annotations using Subsystems Technology (RAST) server version 4.0 (Aziz et al., 2008; Lau, Yin & Chan, 2014). The fundamental properties of the proteins were predicted using ExPASy (Wilkins et al., 1999). Multiple sequence alignment of EasR with other canonical LuxR-type proteins was performed with Clustal OMEGA tool with default parameter settings (available at https://www.ebi.ac.uk/Tools/msa/clustalo/). In addition, a neighbor-joining phylogenetic tree of the *easR* gene with the same canonical LuxR-type proteins was constructed using MEGA-X (version 10.0) (Tamura et al., 2013) whereby the bootstrap has been set to 1,000 repeats. SSpro8 program (available at http://scratch.proteomics.ics.uci.edu/) and Protein Homology/analog Y Recognition Engine (Phyre^2^) (available at http://www.sbg.bio.ic.ac.uk/~phyre2/html/page.cgi?id=index) online tools were used to predict the protein’s secondary and tertiary structures, respectively. To predict functional motifs and domains in EasR, MOTIF (available at https://www.genome.jp/tools/motif/) and InterProScan (available at http://www.ebi.ac.uk/interpro) software were used.

### Construction of recombinant *easR* plasmids

Plasmid DNA for sub-cloning purpose was isolated using QIAprep Spin Miniprep Kit (Qiagen, Hilden, Germany) according to the manufacturer’s instructions. The genomic DNA of strain L1 was extracted using Masterpure™ DNA purification kit (Epicenter; Illumina Inc.,San Diego, CA, USA) as recommended by the manufacturer. The quality of the extracted DNA was checked with Nanodrop Spectrophotometer (Thermo Scientific, Pittsburgh, PA, USA) and agarose gel electrophoresis while DNA quantification was carried out with a Qubit^®^ 2.0 Fluorometer (dsDNA High Sensitivity Assay Kit; Invitrogen, Carlsbad, CA, USA). The extracted genomic DNA was used to amplify the *easR* gene using the following primers: Forward primer, *easR*-F-NdeI (5′ GCAACATATGGAACAGGAGGCAAGCAACTC 3′) and reverse primer, *easR*-R-Bg1II (5′ CAGAGATCTTCAGTCGTCCAGTAATCGTAG 3′). The NdeI and BglII restriction sites were underlined in the primer sequences. To accommodate the frameshift of the recombinant gene sequence, four non-specific bases GCAA were added to the forward primer while three non-specific bases CAG were added to the reverse primer. Polymerase Chain Reaction (PCR) was performed using Q5^®^ High-Fidelity DNA polymerase (NEB, Ipswich, MA, USA). The thermocycler was programed for an initial denaturation step at 98 °C for 30 s, followed by 27 cycles of 98 °C for 10 s, annealing at 57 °C for 30 s, extension at 72 °C for 30 s, a final extension at 72 °C for 2 min and a hold temperature at 4 °C at the end. After PCR, the amplicon was purified using QIAmp^®^ gel extraction kit (Qiagen, Hilden, Germany) before subjecting to ligation into a pGEM^®^-T vector (Promega, Madison, WI, USA) per manufacturer’s instructions. The resultant recombinant plasmid (designated as pGEM^®^-T-*easR*) was chemically transformed into *E. coli* DH5α (Sambrook & Russel, 2001). Blue-white colony screening and colony PCR were performed to allow selection and verification of the recombinants. The *easR* gene was excised from the plasmid by digestion with FastDigest NdeI and BglII (Thermo Scientific, Pittsburgh, PA, USA), followed by gel purification for subsequent ligation with overexpression vector pLNBAD (Lemonnier et al., 2003) digested with the same enzymes. This resultant recombinant plasmid was designated as pLNBAD-*easR*. Sequence verification of the recombinant plasmids was performed by automated Sanger DNA sequencing. The 6.5 kb pLNBAD plasmid was arabinose-inducible to express the target gene. The constructed recombinant plasmids were chemically transformed into *E. coli* TOP10 that harbors pMULTIAHLPROM vector, resulting in another recombinant *E. coli* known as TOP10-pMULTI-pLNBAD-*easR* (Steindler et al., 2008). The pMULTIAHLPROM is a plasmid that carries a synthetic tandem promoter of eight different *luxI* gene promoters (*luxI, cviI, ahlI, rhlI, cepI, phzI, traI and ppuI*) transcriptionally fused to a promoterless lacZ (Steindler et al., 2008). The promoters respond to several different LuxR family proteins and therefore, possess *lux*-boxes which are positively-regulated by the cognate LuxR-family protein in the presence of the cognate AHLs (Steindler et al., 2008). The pLNBAD and pMULTIAHLPROM plasmids maps are shown in Fig. S1.

### Determination of EasR-regulated promoter activities using β-galactosidase assay

The recombinant *E. coli* TOP10 clone which habors both pMULTIAHLPROM and pLNBAD vectors (designated as TOP10-pMULTI-pLNBAD or TOP10-pMULTI-pLNBAD-*easR*) recombinant plasmids were cultured in 10 ml LB broth supplemented with 10 µg/ml tetracycline and 20 µg/ml chloramphenicol at 30 °C with agitation at 250 rpm. An aliquot of the overnight bacterial cultures was inoculated into 10 ml of sterile fresh LB broth supplemented with appropriate antibiotics to produce a starting OD_600_ ~0.02. When indicated, AHLs (C4-HSL, C6-HSL, *N*-decanoyl homoserine lactone (C10-HSL) and *N*-dodecanoyl homoserine lactone (C12-HSL)) were added to clones TOP10-pMULTI-pLNBAD or TOP10-pMULTI-pLNBAD-*easR* at a final concentration of 100 µM. Two sets of cultures were prepared for each sample. One set of clones was induced by adding one mM of L-arabinose while another set was grown without any inducer. All cultures were grown under the same conditions until mid-log phase was achieved (OD_600_ ~0.4–0.6) before they were placed on ice. Following this, two ml aliquot of the bacterial cultures was centrifuged at 3,500×*g* for 10 min and the resulting cell pellet was resuspended in two ml chilled Z buffer (0.06 M Na_2_HPO_4_·7H_2_O, 0.04 M NaH_2_PO_4_·H_2_O, 0.01 M KCl, 0.001 M MgSO_4_, 0.05 M β-mercapthoethanol, pH7). The OD_600_ of the resuspended cells was measured spectrophotometrically with Z buffer as a blank. To permeabilize the cells, 1 ml of cells in Z buffer was mixed with 100 μl chloroform and 50 μl of 0.1 % w/v sodium dodecyl sulfate, vortexed and equilibrated for 5 min in a 28 °C heat block. The β-galactosidase assay was initiated by the addition of 0.2 ml *ortho*-Nitrophenyl-β-galactoside (ONPG, 4 mg/ml) as the substrate into the lysate. The tubes were vortexed and incubated at 28 °C. The color changes of the tubes were observed until sufficient yellow color (as the color of LB broth) had developed. The reaction was then stopped by the addition of 0.5 ml of 1 M sodium carbonate, Na_2_CO_3_. The exact time taken from the addition of ONPG to the stopping of the reaction with Na_2_CO_3_ was recorded. The mixture was centrifuged at maximum speed for 5 min to remove cellular debris and chloroform (Chan, 2011). The OD at 420 nm and at 550 nm for each tube was recorded (blanked against the same mixture but without cells). The units of enzymatic activity, expressed as Miller units, were calculated using the following equation (Miller, 1972):

$${\rm Miller \; Units} = 1,000 \times [({\rm OD_{420}} - 1.75 \times {\rm OD_{550}})]/({\rm T} \times {\rm V} \times {\rm OD_{600}})$$

where:

• OD_420_ and OD_550_ were read from the reaction mixture

• OD_600_ reflected cell density in the washed cell suspension

• T = time of the reaction, in min

• V = volume of culture used in the assay, in ml

Mean values of nine separate independent experiments were calculated. The experiment was repeated with constructs harboring pMULTIAHLPROM and pLNBAD-*easR* (designated as TOP10-pMULTI-pLNBAD or TOP10-pMULTI-pLNBAD-*easR*).

### Statistical analysis

All values were expressed as the mean ± SD of nine observations. Statistical analyses were performed using Student’s *t*-test. Values are the mean ± SD of nine separate independent experiments. For all analyses, a *P* value ≤ 0.05 was considered statistically significant at a confidence interval 95%.

## Results

### Nucleotide sequence and bioinformatics analysis

**Figure 1:**
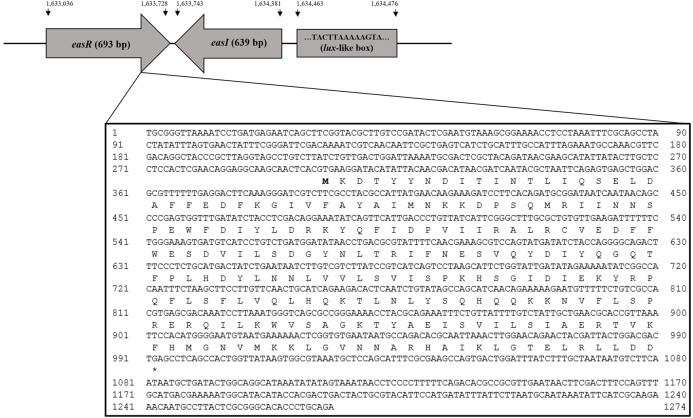
Organization of *easR*, *easI* and *lux*-liked box in *E. asburiae* strain L1 genome. The nucleotide sequences of *easR* gene and its flanking sequences are enlarged.

**Figure 2:**
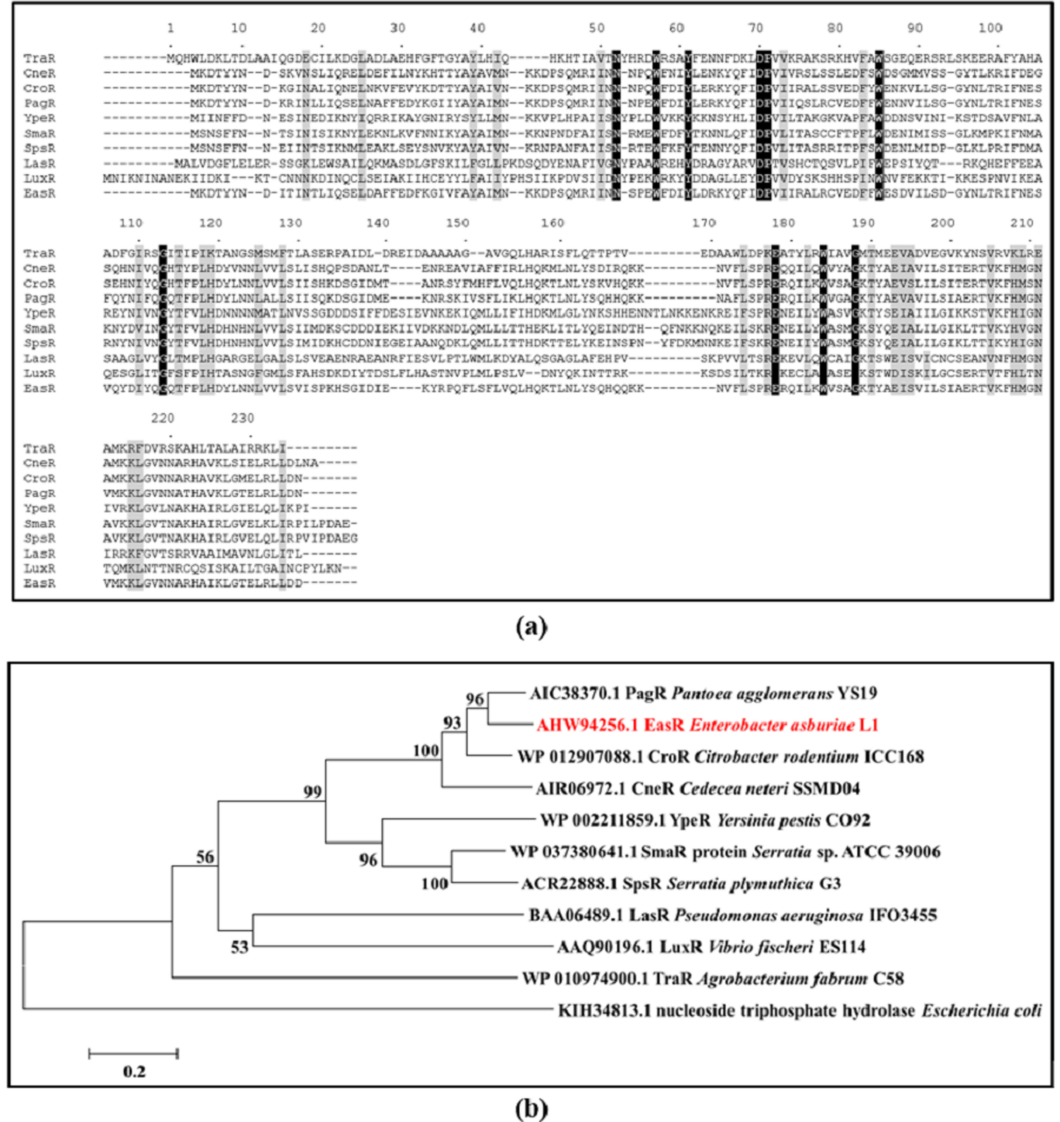
(A) CLUSTAL O (1.2.0) multiple sequence alignment and (B) Phylogenetic tree of *E. asburiae* strain L1 EasR protein. (A) CLUSTAL O (1.2.0) multiple sequence alignment of *E. asburiae* strain L1 EasR protein with nine canonical QS LuxR-type proteins. Absolutely conserved residues are given a black background while those that are highly similar among the sequences are denoted by a gray background. GenBank accession numbers in parentheses: TraR *Agrobacterium fabrum* C58 (AAK91098.1), CneR *Cedecea neteri* SSMD04 (AIR06972.1), CroR *Citrobacter rodentium* ICC168 (CBG89690.1), PagR *Pantoea agglomerans* YS19 (AIC38370.1), YpeR *Yersinia pestis* CO92 (AAF21289.1), SmaR *Serratia* sp. ATCC 39006 (CAB92554.1), SpsR *Serratia plymuthica* G3 (ACR22888.1), LasR *Pseudomonas aeruginosa* IFO3455 (BAA06489.1), LuxR *Enterobacter cloacae* complex sp. FDAARGOS 77 (AVG33608.1), and LuxR *Vibrio fischeri* ES114 (AAW87995.1). TraR residue numbering is shown above the alignment as reference. (B) Phylogenetic tree showing the evolutionary distances between the transcriptional regulator, EasR of strain L1 (red word) with the other canonical QS LuxR-type proteins. The tree was generated using neighbor-joining algorithm and was drawn to scale, with branch lengths to show the evolutionary distances. The bootstrap values as percentage of 1,000 replications are given as numbers at the nodes. The horizontal bar indicates evolutionary distance as 0.2 change per nucleotide position. Nucleoside triphosphate hydrolase of *Escherichia coli* represents an outgroup.

Analysis of strain L1 genome revealed an open reading frame coding for a putative *luxR* homolog, hereafter named *easR* (GenBank accession number AHW94256.1). Based on the NCBI database (https://www.ncbi.nlm.nih.gov/), the 693 bp *easR* (Fig. S2) encodes a protein with 230 amino acids and is located in between 1,633,036 and 1,633,728 of strain L1 complete genome (Lau, Yin & Chan, 2014). Prediction from ExPASy server (Wilkins et al., 1999) showed that the molecular mass (Mr) of EasR is 27.04 kDa while the isoelectric point (pI) is 7.02. The gene is convergently transcribed with respect to *easI* with an intergenic region of 14 bp. Figure 1 outlines the organization of *easR*, *easI* and *lux*-liked box in strain L1 genome. Multiple sequence alignment of EasR and other canonical LuxR-type proteins (Fig. 2A) revealed the presence of conserved sites among these ten LuxR-type proteins, namely residues N52, W57, Y61, D70, P71, W85, G113, E178, W184, G188 (TraR residue numbering is used as a reference). In addition, phylogenetic analysis was performed on the protein sequence of *easR* gene with other canonical LuxR-type proteins, retrieved from the genomes available in NCBI database (Fig. 2B). The LuxR tree indicated that EasR of strain L1 shared the highest similarity with LuxR (Pairwise alignment: EasR = 99.6% similarity of amino acid residues) of *Enterobacter* genus, followed by PagR of *Pantoea agglomerans* YS19, CroR from *Citrobacter rodentium* ICC168 and CneR from *Cedecea neteri* SSDM04 (Pairwise alignment: EasR = 93.5%, 87.4% and 81.9% similarity of amino acid residues, respectively).

Using the SSpro8 program that predicts the secondary structure of a protein, it appears that the molecule that made up EasR protein consists of ten distinct α helices: α1 3→25, α2 51→59, α3 67→74, α4 91→102, α5 133→157, α6 163→165, α7 170→179, α8 185→192, α9 196→209, α10 215→225 and five ß sheets: ß1 30→35, ß2 45→47, ß3 79→81, ß4 109→113 ß3 120→126 (Fig. S3A). The predicted tertiary structure of EasR is illustrated in Fig. S3B. The colored ribbon diagram indicates the N- to C-terminal positions of residues within EasR sequence.

**Figure 3:**
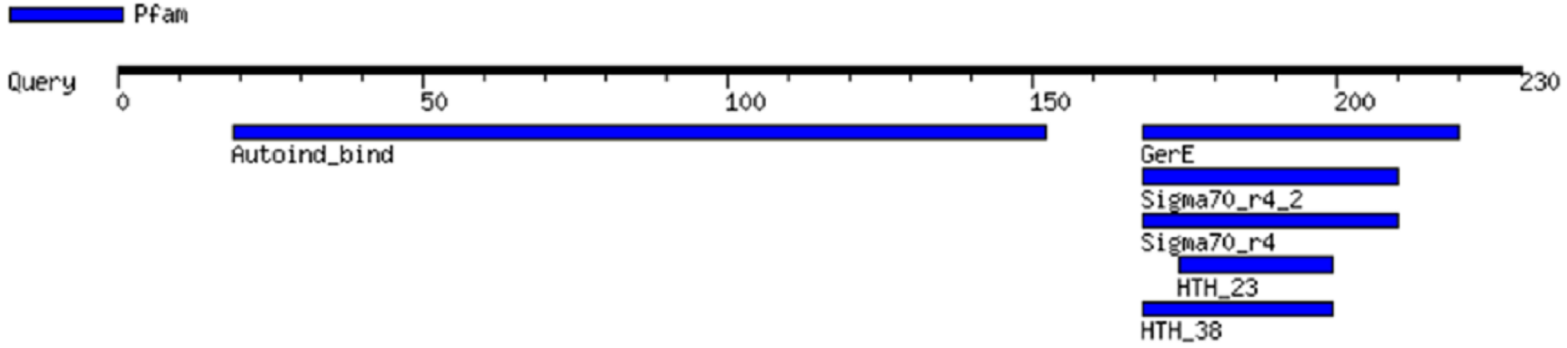
*E. asburiae* strain L1 EasR functional domain analysis using MOTIF. Analysis revealed the presence of the two principal conserved domains in LuxR: an N-terminal AHL-binding domain and a C-terminal DNA binding HTH domain.

MOTIF revealed the presence of the two principal conserved domains in LuxR: an N-terminal AHL-binding domain and a C-terminal DNA binding HTH domain (Fig. 3). The analysis by MOTIF indicated that the inducer binding site and activator site of EasR extended from amino acid residues 19 to 152 and 168 to 220, respectively. MOTIF also predicted that EasR possesses homeodomain-like domain, which is a protein structural domain that binds DNA. Residues 168 to 199 form the HTH, which is a highly conserved region characterizing the LuxR family (Fuqua, Winans & Greenberg, 1996; Zaghlool & Al-Khayyat, 2015). Besides, a significant sequence similar to region 4 of the sigma factor belonging to RNA polymerase (extended from amino acid 168 to 210) was found at the C-terminal region of the EasR. This motif is a HTH-containing region that could recognize the −35 sequences of promoters of LuxR-regulated genes (Kahn & Ditta, 1991; Zaghlool & Al-Khayyat, 2015). The position, the independent *e*-value and the recognition sequence of the functional motifs in EasR as predicted by MOTIF, are listed in Table S2.

Additionally, InterProScan was used to further analyze the motifs found in EasR, as predicted by MOTIF. Analysis showed the presence of four motifs; (i) transcriptional factor LuxR-like autoinducer-binding domain (IPR036693), (ii) winged helix-like DNA-binding domain (IPR036388), (iii) transcriptional regulator LuxR, C-terminal (IPR000792), and (iv) signal transduction response regulator, C-terminal effector (IPR016032; a fragment of the two-component signal transduction system; an unrelated motif) (Zaghlool & Al-Khayyat, 2015). InterProScan analysis also showed that the C-terminal dimerization of EasR encompassed amino acid residues S180, G182, T184, A215, R216, H217, and K220.

### Determination of EasR-regulated promoter activities using β-galactosidase assay

**Figure 4:**
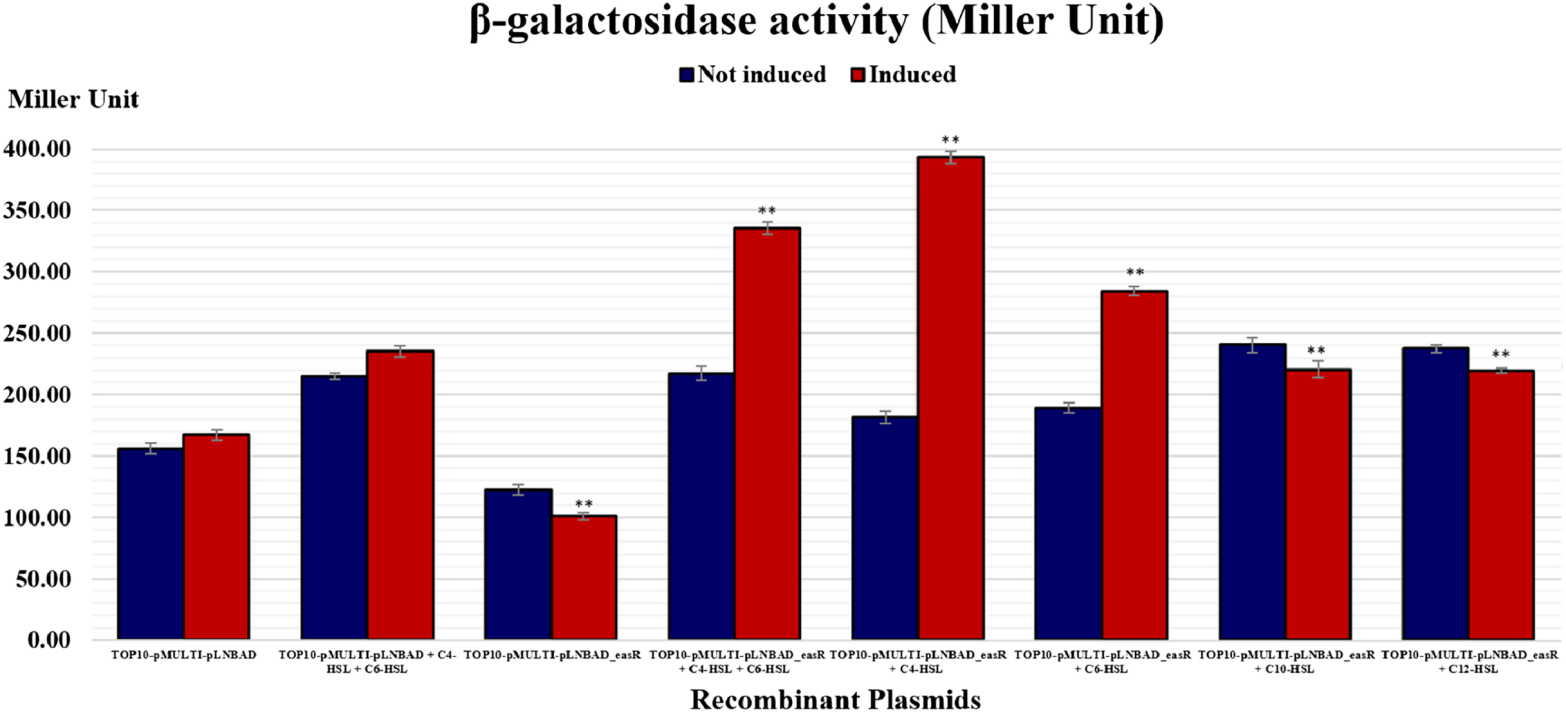
Histogram reporting EasR*-*regulated promoter β-galactosidase activity of *E. coli* TOP10 harboring pMULTIAHLPROM and pLNBAD (TOP10-pMULTI-pLNBAD) or pLNBAD-*easR* (TOP10-pMULTI-pLNBAD-*easR*) in the presence or absence of 100 µM different AHLs (C4-HSL, C6-HSL, C10-HSL and C12-HSL). All measurements were expressed in Miller units and indicated as mean values of nine separate independent experiments. Bars: Standard errors of the mean. The significance levels have been displayed on the histogram, ** for *p* < 0.01.

It was postulated that if pMULTIAHLPROM is harbored by *E. coli* TOP10 that overexpressed a LuxR homolog, that is, EasR, this can lead to transcription initiation from one or more of the *luxI*-family gene promoters. Therefore, the promoter activities can then be detected using β-galactosidase assay. In this study, pLNBAD (empty vector) and pLNBAD-*easR* recombinant were successfully introduced into the *E. coli* TOP10 that harbored pMULTIAHLPROM plasmid, respectively. The recombinants are designated as TOP10-pMULTI-pLNBAD and TOP10-pMULTI-pLNBAD-*easR*. The *lacZ* activities were determined by adding different chain length of exogenous AHLs. Results show that the β-galactosidase activities of cells harboring TOP10-pMULTI-pLNBAD-*easR* was significantly higher in the presence of 100 µM exogenous AHLs (C4-HSL, C6-HSL, C10-HSL and C12-HSL). Our findings also revealed that C4-HSL could activate the β-galactosidase activities of cells harboring TOP10-pMULTI-pLNBAD-*easR* at the highest intensity, which is approximately three times higher in Miller units when compared with the absence of AHLs. Surprisingly, when both exogenous C4-HSL and C6-HSL were present together, the intensity of the β-galactosidase activities of cells harboring TOP10-pMULTI-pLNBAD-*easR* was not as high as in the presence of C4-HSL alone. Table 1 and Fig. 4 also show that there is a slight increase in β-galactosidase activity for induced samples with long chain AHLs (i.e., C10-HSL and C12-HSL). However, the intensity of the activities is the lowest among all other AHLs. A point worth noting, the intensity of the β-galactosidase activities of cells harboring TOP10-pMULTI-pLNBAD-*easR* in response to both of the added long chain AHLs (C10-HSL and C12-HSL) are more or less the same. Besides, the β-galactosidase activities of TOP10-pMULTI-pLNBAD-*easR* plasmid were also significantly higher when compared with TOP10-pMULTI-pLNBAD empty vector control in the presence or absence of AHLs.

**Table 1:** β-galactosidase activity (in Miller units) of TOP10-pMULTI-pLNBAD and TOP10-pMULTI-pLNBAD*-easR* under different conditions.

Recombinant clones	Miller units (Mean ± SD)
Top10-pMULTI-pLNBAD (Control)	
Not induced	155.95 ± 4.49
Induced	167.15 ± 3.93
Not induced + 100 µM C4-HSL + 100 µM C6-HSL	214.72 ± 2.73
Induced + 100 µM C4-HSL + 100 µM C6-HSL	235.12 ± 4.57
Top10-pMULTI-pLNBAD *-easR*	
Not induced	122.24 ± 4.07
Induced	100.93 ± 3.05
Not induced + 100 µM C4-HSL + 100 µM C6-HSL	217.03 ± 5.81
Induced + 100 µM C4-HSL + 100 µM C6-HSL	335.50 ± 5.02
Not induced + 100 µM C4-HSL	181.52 ± 5.09
Induced + 100 µM C4-HSL	393.36 ± 4.91
Not induced + 100 µM C6-HSL	189.18 ± 4.41
Induced + 100 µM C6-HSL	284.27 ± 3.61
Not induced + 100 µM C10-HSL	240.34 ± 6.22
Induced + 100 µM C10-HSL	220.46 ± 6.69
Not induced + 100 µM C12-HSL	237.36 ± 3.36
Induced + 100 µM C12-HSL	219.48 ± 1.92

## Discussion

For the past decade, a large family of AHL-based QS system has been characterized, each resembling the *luxI* and *luxR* homologs of *Vibrio fischeri* (Zhang et al., 2002). The LuxI protein is required for the synthesis of the autoinducer, while the LuxR protein is the transcriptional regulator that binds to autoinducer to form a complex, which, in turn binds to the promoter of the QS-regulated genes (Engebrecht & Silverman, 1984).

LuxR-type proteins are approximately 250 amino acids long and consist of two domains. The N-terminal fragment, usually made of two-third of the amino acid residues, binds its cognate AHLs as ligands. In an early study by Hanzelka & Greenberg (1995), an overexpression of N-terminal domain of LuxR of *V. fischeri* in *E. coli* demonstrated that the fragment was sufficient to sequester its autoinducers. This domain has been shown to be responsible in multimerization of the full-length protein (Choi & Greenberg, 1992). In the absence of AHL, the cognate LuxR protein does not fold correctly and undergoes rapid degradation. However, autoinducer binding to LuxR produces a stable complex that could elicit a downstream signal transduction cascade (Hanzelka & Greenberg, 1995). This has been demonstrated in *Agrobacterium tumefaciens*, in which the purified wild-type TraR (LuxR homolog) was reported to be in a predominantly dimeric configuration in the presence of AHL. The protein dimers tend to dissociate into its monomer form if the AHL signal was removed (Qin et al., 2000; Zhu & Winans, 2001). The N-terminal domain of most LuxR homologs constitutes a high percentage of hydrophobic and aromatic residues. Likewise, in this study, EasR was found to possess high numbers of hydrophobic residues such as isoleucine, leucine, phenylalanine, and tyrosine. Many QS bacteria have evolved to possess transcriptional regulators that are genetically conserved among LuxR family, and therefore, have high specificity to their cognate signal (Zhang et al., 2002).

On the other hand, the C-terminal domain of LuxR proteins possesses the specific DNA binding domain with the four HTH motifs (Fig. S3). Choi & Greenberg (1991) reported that the overexpressed C-terminal fragment of *V. fischeri* was capable to function alone as a transcriptional activator. Besides, the synergistic binding of the C-terminal domain and RNA polymerase facilitates the latter to bind to the target promoter region (Stevens, Dolan & Greenberg, 1994). In fact, using mutagenesis studies, the residues that constitute the *V. fischeri* LuxR C-terminal domain were found to be important in the interaction with RNA polymerase (Egland & Greenberg, 2001).

Interestingly, unlike LuxI homologs, it was found that LuxR-type proteins share low similarities (18–25 %) among QS bacteria (Whitehead et al., 2001; Zhang et al., 2002). Out of nine conserved residues, six are located in the N-terminal domain and the remaining three are in the C-terminal. EasR was found to possess all conserved residues (W57, Y61, D70, P71, W85, G113, E178, W184, G188), which are present in at least 95% of LuxR-type proteins (Whitehead et al., 2001; Zhang et al., 2002). Even though EasR was found to share low similarities to other LuxR proteins (Fig. 2A), it was clustered closely with LuxR from *Enterobacter cloacae* complex sp. FDAARGOS 77, followed by PagR from *P. agglomerans* YS19, CroR from *C. rodentium* ICC168 and CneR from *C. neteri* SSDM04 as illustrated in the phylogenetic tree (Fig. 2B). It may indicate that these Proteobacteria share a similar mechanism in expression and activation of LuxR, even though their QS regulators are responsible for different target genes.

A point worth noting, *easR* gene showed high sequence identity to the *luxR* gene of other *Enterobacter* species. BLAST search of the EasR amino acid sequence revealed a high similarity with many members of the *E. cloacae* complex, an important group of nosocomial pathogens (Lau, Yin & Chan, 2014). Unfortunately, there is very little information associated with AHL-type QS mechanism in *Enterobacter* spp. especially in unraveling the complex mechanisms that contribute to the pathogenicity of different *Enterobacter* spp. Further studies are indeed required to highlight the association of QS with pathogenicity in *Enterobacter* spp.

Studies show that the genetic organization of both LuxI and LuxR proteins is as diverse as their functions. Some LuxI and LuxR pairs are transcribed convergently while others in a divergent way. Both *easI* (*luxI* homolog) and *easR* (*luxR* homolog) are organized in a convergent manner, similar to other Enterobacter species and in most Gammaproteobacteria (Gray & Garey, 2001). In bacteria which possess such convergent organization, it was reported that the expression of *luxI* mostly is not under the control of *luxR* (Atkinson et al., 1999). However, in this study, more experimental data is needed to establish the relationship between the regulatory roles of EasI and EasR.

In this study, the transcriptional regulator, *easR* gene of *E. asburiae* strain L1 was cloned to the downstream of a PBAD promoter in pLNBAD vector, followed by transformation into *E. coli* TOP10, together with pMULTIAHLPROM vector. The pLNBAD is a vector that harbors an inducible PBAD promoter which is positively and also negatively-regulated by the products of the *araC* gene (Schleif, 2010). In the presence of L-arabinose, AraC forms a complex with L-arabinose, allowing transcription to begin. On the other hand, the pMULTIAHLPROM is a pMP220-derived plasmid which carries a synthetic tandem promoter of eight different *luxI* gene promoters (*luxI, cviI, ahlI, rhlI, cepI, phzI, traI and ppuI*) transcriptionally fused to a promoterless *lacZ* gene. These promoters were chosen over other promoters as all of them are known to carry *lux*-boxes which are positively-regulated by the cognate LuxR-family protein in the presence of the cognate AHL (Steindler et al., 2008). In this assay, it is important to take note that *E. coli* TOP10 was chosen over *E. coli* DH5α due to its capability of transporting L-arabinose, but not metabolizing it, and thus, ensure the level of L-arabinose is constant inside the cells and not decreasing over time.

To determine the functionality of *easR* in *E. asburiae* strain L1, the recombinants were then subjected to β-galactosidase assay either in the presence or absence of exogenous AHLs. The gene β-galactosidase, encoded by *lacZ*, hydrolyzes β-D-galactosides to allow the bacteria to grow on carbon sources (e.g., lactose), by cleaving it into simple sugars (i.e., glucose and galactose), so that it can provide energy to sustain the growth of the bacteria cells. In this study, the β-galactosidase assay utilized ONPG in place of lactose as the substrate and cleaved to yield galactose. At the same time, cleavage of ONPG by β-galactosidase also releases *o*-nitrophenol with yellow color and absorbs at 420 nm (Miller, 1972). The reading at 420 nm is a combination of absorbance by *o*-nitrophenol and light scattering by cell debris. The increase in absorbance at 420 nm would be a reflection of β-galactosidase activity in the cells. This assay was also employed by Egland & Greenberg (2000) in studying LuxR and *lux* box in *V. fischeri*. It was postulated that if the overexpressed *easR* is a functional transcriptional regulator, it could bind complementary to both cognate C4-HSL and C6-HSL, hence, possibly initiate transcription from one or more of the *luxI*-family gene promoters present in pMULTIAHLPROM. In addition, another two long-chain AHLs (C10-HSL and C12-HSL) were included to test the specificity of EasR response to initiate transcription from any of the *luxI*-family gene promoters present in pMULTIAHLPROM apart from the cognate AHLs by *E. asburiae* strain L1. When ONPG is in excess over the enzyme in a reaction, the production of *o*-nitrophenol per unit time is proportional to the concentration of β-galactosidase; thus, the formation of yellow color can be used to determine the enzyme concentration.

The functionality of the *easR* was validated by the addition of exogenous AHLs. Higher β-galactosidase activities were detected for cells harboring TOP10-pMULTI-pLNBAD-*easR* in the presence of exogenous AHLs compared to the one without AHLs. On top of that, β-galactosidase activities of cells harboring TOP10-pMULTI-pLNBAD-*easR* with the addition of C4-HSL was present in a higher amount than C6-HSL. This was in agreement with the previous hypothesis that the former AHL may possess a more important role in executing the physiological functions of the cells or expression of virulence factors (Lau et al., 2018). Surprisingly, a combination of C4-HSL and C6-HSL were not able to elicit higher activity than C4-HSL alone. In this study, EasR was exposed to high concentration of AHLs in the presence of both C4-HSL and C6-HSL (Total concentration; 200 μM), therefore it may affect the formation of the EasR-AHL complex. Besides, C6-HSL may not form the most stable conformation with EasR and hence there is a preference of EasR to bind to C4-HSL. Nevertheless, more experimental data is needed to verify the findings.

Furthermore, in previous study, *E. asburiae* strain L1 was found to produce C4-HSL as the major AHL and C6-HSL as the minor AHL (Lau et al., 2018). Therefore, in the current study, it was predicted that C4-HSL will elicit higher β-galactosidase activities than C6-HSL alone. This was in agreement with the previous hypothesis. In many cases, *luxR* can both activate and suppress the *luxI* gene (Lequette et al., 2006). A study by Li et al. (2019) suggested that LuxR protein might regulate AHL expression either positively or negatively. When the AHL is present alone, it might be positively or negatively regulate the expression of the *luxI* gene and expressing more or lesser C4-HSL or C6-HSL. However, when both AHLs are present together, the regulation might act differently. This might be the reason why the combination of C4-HSL and C6-HSL in this study did not elicit higher activity than C4-HSL alone. Whether EasR acts as an activator or repressor remains a question and requires further validation in the future.

Besides, we also found a slight increase in β-galactosidase activity for induced samples with long chain AHLs (i.e., C10-HSL and C12-HSL). Although the statistical analysis has shown a significant increase in the β-galactosidase activity of the induced samples with long chain C10-HSL and C12-HSL, the miller units in between the induced and non-induced samples are in fact quite similar. Lindsay & Ahmer (2005) mentioned that there are some bacterial species of the genera *Escherichia*, *Salmonella*, and *Klebsiella* which are unique in their cell-signaling process, in which these bacteria possess a LuxR homolog, SdiA, but they do not possess a LuxI homolog or any other enzyme family that can synthesize AHLs. Using SdiA, these bacteria such as *E. coli* and *Salmonella enterica* manage to detect and bind to the QS signal AHLs produced by other bacteria (Kendall & Sperandio, 2014; Lee et al., 2009; Lindsay & Ahmer, 2005). A study by Smith et al. (2008) has shown that SdiA could respond to C10-HSL and C12-HSL at a concentration as low as 1 μM. In our study, we have used 100 μM AHL for the β-galactosidase assay and this concentration should be sufficient to be detected by SdiA (Smith et al., 2008). Therefore, we postulated that the β-galactosidase activities obtained with C10-HSL and C12-HSL could be due to the presence of SdiA in the *E. coli* TOP10. However, this requires further validation. Further studies will be performed using the *E. coli* strain mutated in SdiA to prove that EasR could recognize both long chain C10-HSL and C12-HSL.

There are strong evidences that demonstrated the ability of various LuxR homologs to respond to non-cognate AHL molecules. Such interactions were exemplified in some bacterial species such as *Beneckea harveyi*. *V. fischeri*, *C. violaceum*, and *Aeromonas hydrophila* (Greenberg, Hastings & Ulitzur, 1979; McClean et al., 1997; Schaefer et al., 1996; Swift et al., 1999). These non-cognate AHLs from other bacterial species may activate or suppress the function of transcriptional regulators. Additionally, cell-to-cell communication was reported to occur between opportunistic human pathogens *Burkholderia cepacia* and *Pseudomonas aeruginosa*, possibly in regulating virulence factors production that could contribute to pneumonia in immunocompromised individuals (McKenney, Brown & Allison, 1995). It was also recently found that some LuxR-type proteins have evolved to respond to molecules or signals other than AHLs such as plant-based molecules (Patel et al., 2013).

Most of the LuxR homologs appear to function as positive regulators in a multitude of cellular behaviors. However, several LuxR-type regulators work on the contrary side. This is demonstrated by EsaR of *P. stewartii*, in which the protein is active in the absence of any AHL, and its activity is blocked by its cognate AHLs. It was reported that EsaR represses transcription of its own gene but does not affect the expression of *esaI* (Tsai & Winans, 2010). In fact, EsaR has been shown to act as a repressor in the synthesis of exopolysaccharide and this negative activity was alleviated by the addition of exogenous AHLs (Von Bodman, Majerczak & Coplin, 1998). LuxR proteins that act as repressors have also been demonstrated in *Serratia* species, such as SpnR of *S. marcescens* SS-1 (Horng et al., 2002), SmaR of *Serratia* sp. ATCC 30096 (Slater et al., 2003) and SprR of *S. proteamaculans* B5a (Christensen et al., 2003). Whether EasR acts as an activator or repressor remains a question and requires further validation in the future.

## Conclusion

This study is the first report documenting the cloning and characterization of transcriptional regulator, *luxR* homolog from *E. asburiae*. The functionality and specificity of EasR protein in response to different AHL signaling molecules to activate gene transcription from QS target promoters was determined via β-galactosidase assay. In this work, we have identified the AHLs sensed by EasR in *E. asburiae*. On a final note, future studies will be focused on the genome-wide comparative transcriptomics by knocking out both *easI* and *easR* genes of *E. asburiae* strain L1, in the hope to diversify the knowledge of the possible roles played by QS in *E. asburiae*.

## Supplemental Information

10.7717/peerj.10068/supp-1 Supplemental Information 1 Supplementary Figures. The secondary and tertiary structure of EasR and the vectors map.

10.7717/peerj.10068/supp-2 Supplemental Information 2 Supplementary Tables. Bacterial strains and plasmids used in this study and the functional motifs in EasR predicted by MOTIF software.

## Additional Information and Declarations

### Data Availability

The following information was supplied regarding data availability: The raw measurements are available in the Supplemental Files.
