# Supplementary material for: Functional characterization of quorum sensing LuxR-type transcriptional regulator, EasR in Enterobacter asburiae strain L1
Source: PeerJ. 2020 Oct 21;8:e10068. doi: 10.7717/peerj.10068 (PMC7585371; doi:10.7717/peerj.10068)
Supplement: Supplemental Information 1 — The secondary and tertiary structure of EasR and the vectors map. [file peerj-08-10068-s001.docx]

**Supplementary Fig. S1.**


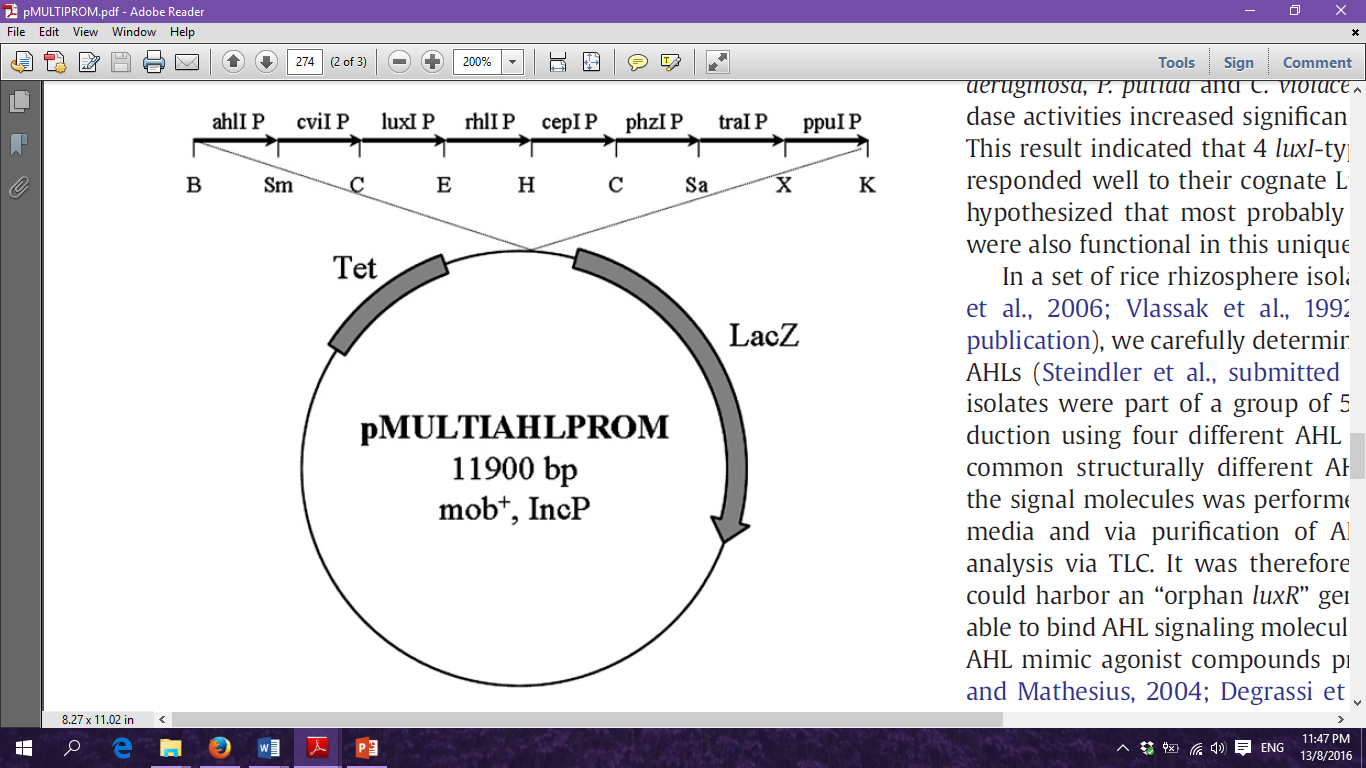


**(a)**


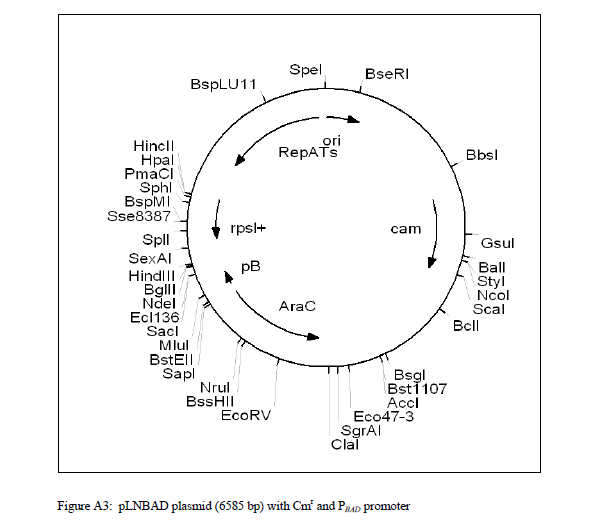


**(b)**

**Fig. S1.** (a) Vector map of pMULTIAHLPROM plasmid (11900 bp) with Tet^R^ (Steindler et al., 2008); (b) Vector map of pLNBAD plasmid (6585 bp) with Cm^R^ and PBAD promoter (Lemonnier et al., 2003).

**Supplementary Fig. S2.**

**
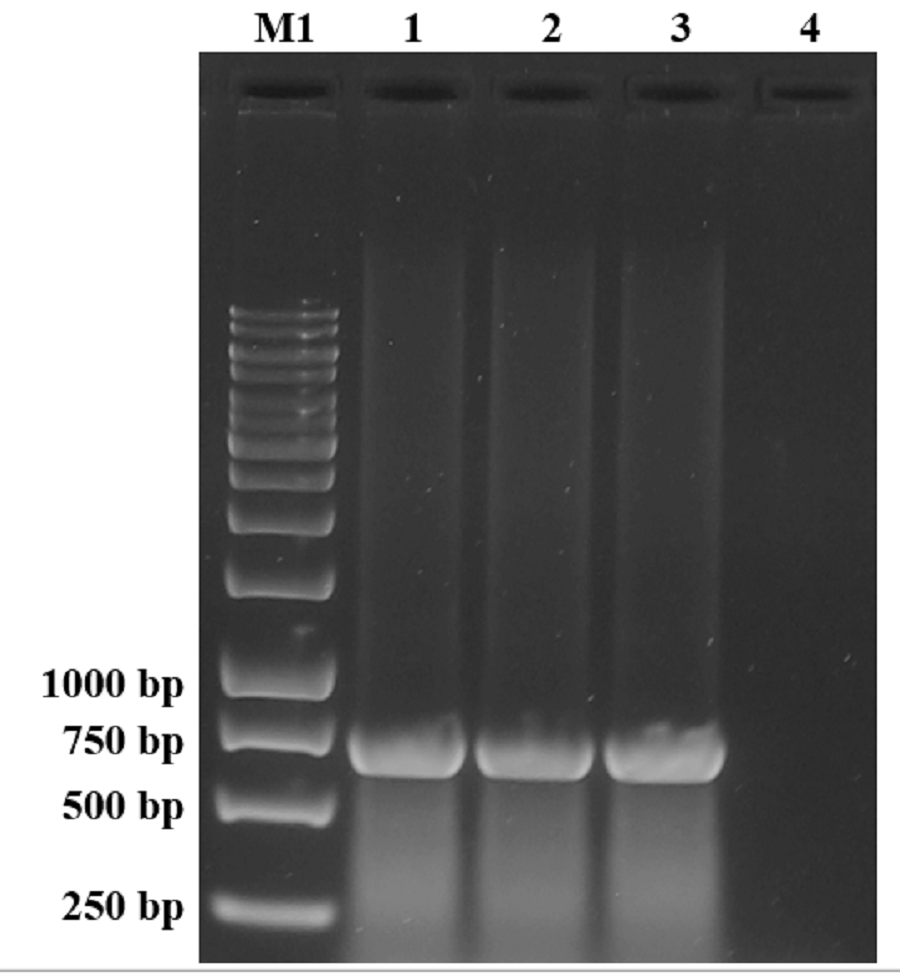
**

**Fig. S2.** Agarose gel electrophoresis of the PCR-amplified *easR* gene from the genomic DNA of strain L1. Lane M1, 1 kb DNA ladder (Fermentas, Thermo Fisher Scientiﬁc, USA); Lanes 1 to 3, 693 bp amplicons in replicates; Lane 4, negative control.

**Supplementary Fig. S3.**

**Predicted Secondary Structure:**

CCHHHHHHHHHHHHHHHHHHHHHHHCCCCEEEEEECCCCCCCCCEEECCCHHHHHHHHHCCCCCCCHHHHHHHHCCCCEEECCCCCCCCCHHHHHHHHHHHHCCCCCCEEEEECCCCCCEEEEEEECCCCCCHHHHHHHHHHHHHHHHHHHHHHHHHCCCCCHHHCCCCHHHHHHHHHHCCCCCHHHHHHHHCCCHHHHHHHHHHHHHHCCCCCHHHHHHHHHHHCCCCC


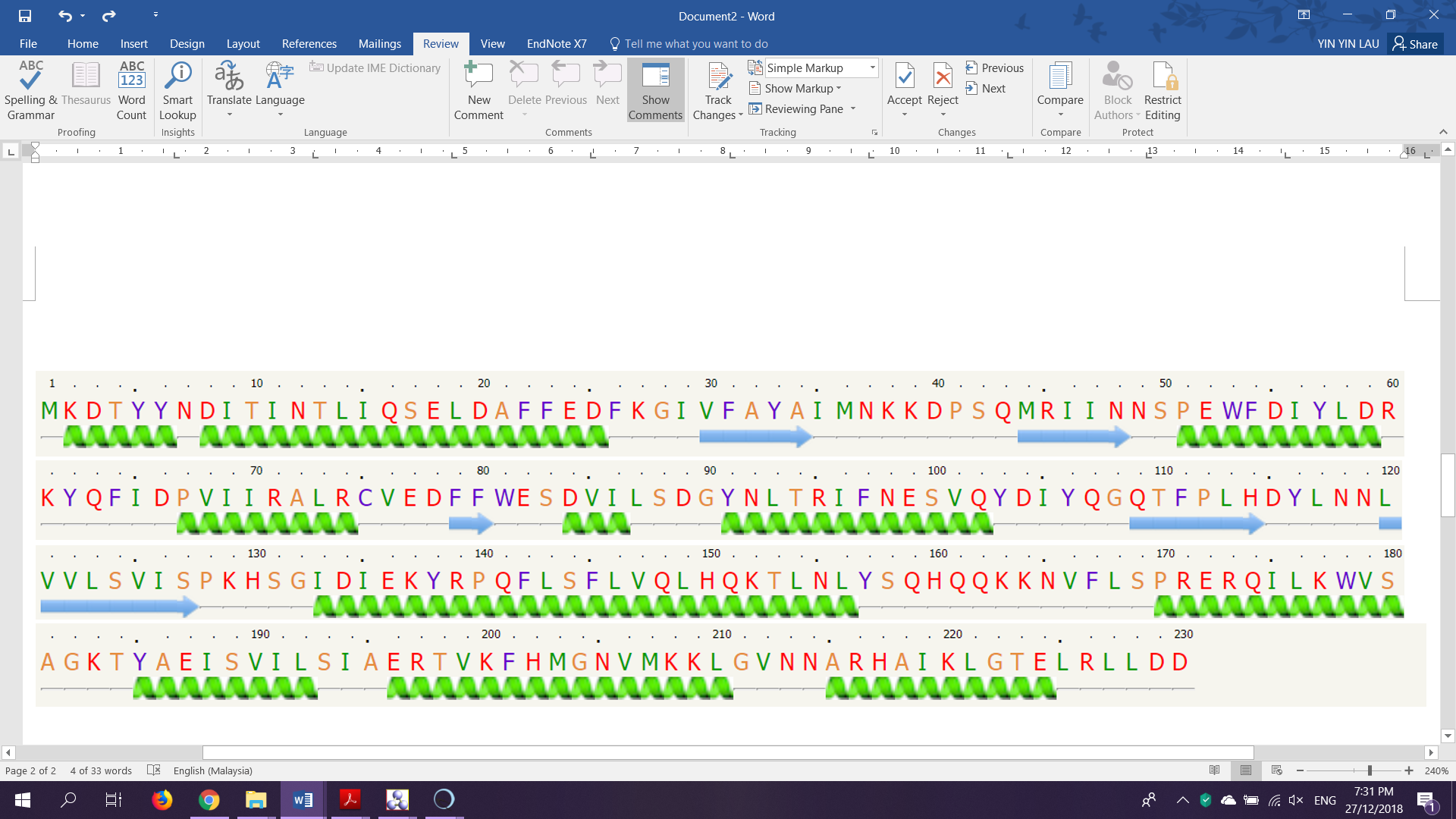


**(a)**


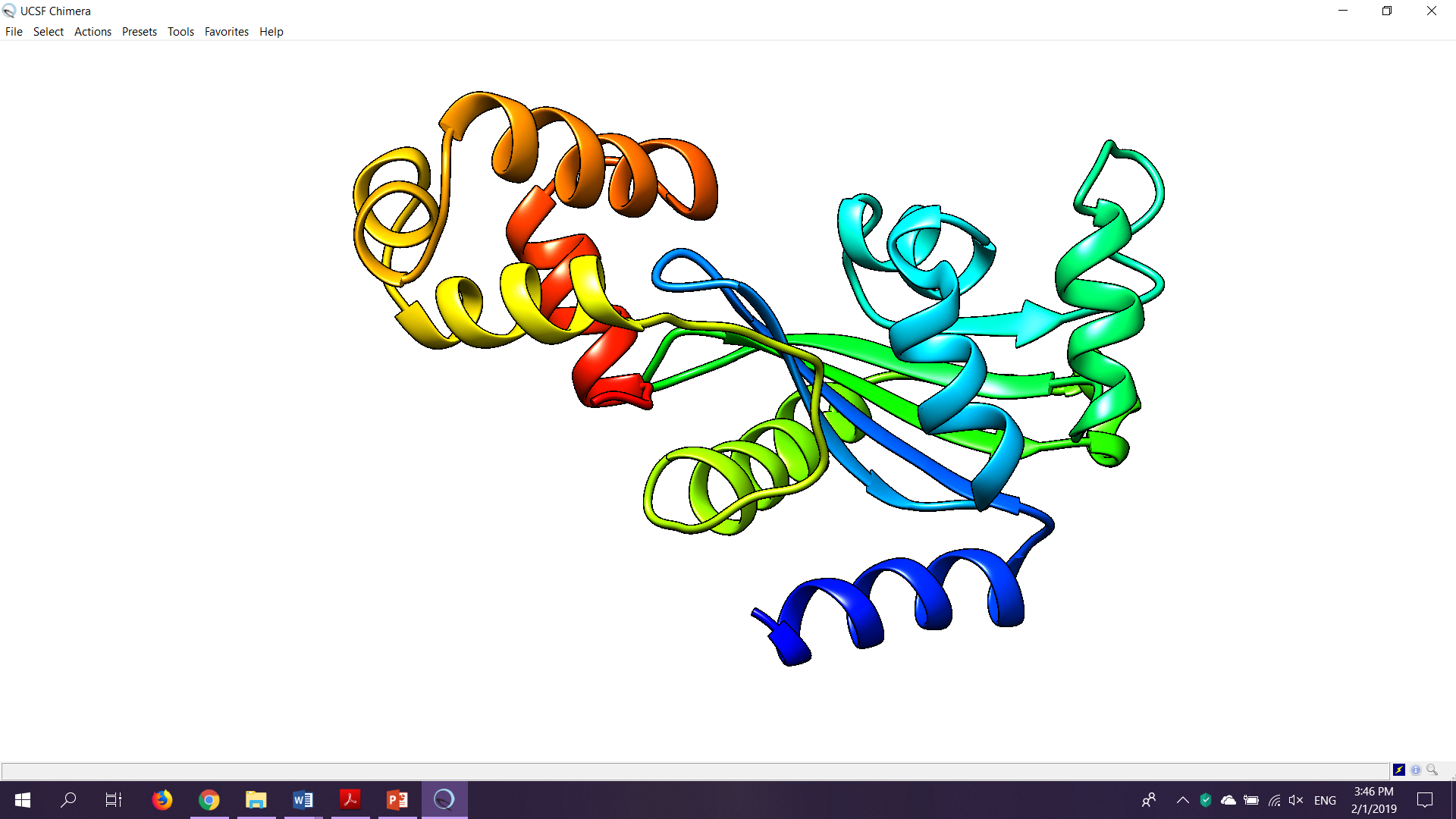


**(b)**


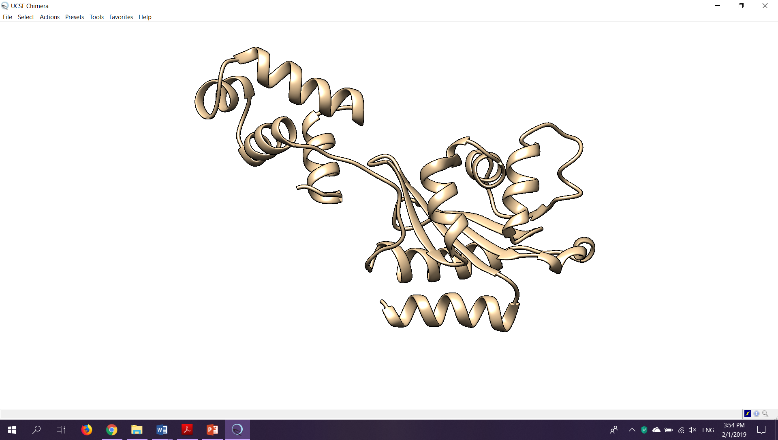

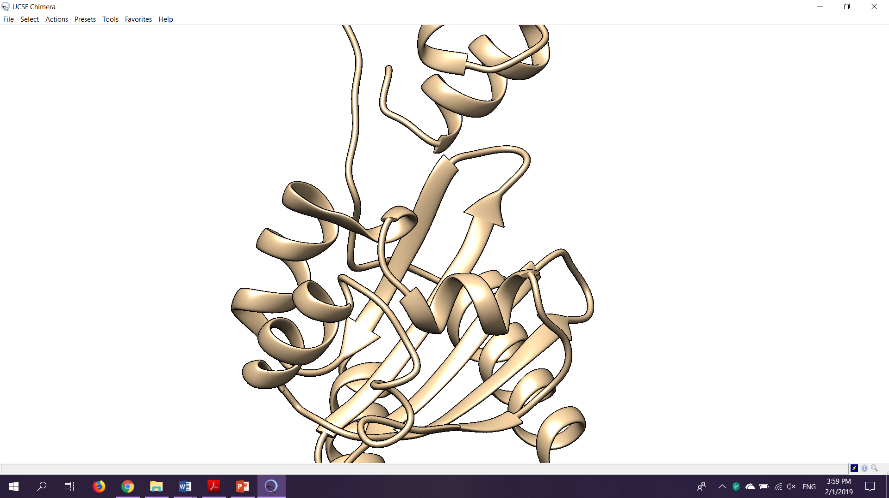
**Fig. S3.** (a) Secondary structure of EasR as predicted by SSpro8. H: alpha-helix, E: extended strand, C: the rest. (b) EasR tertiary structure is composed of ten distinct α helices ( ) and five ß sheets ( ) connected by loops as predicted by Phyr^2^. A ribbon diagram, colored from blue to red, indicates the N‐ to C‐terminal positions of residues within the sequence.
