# Supplementary material for: Functional characterization of quorum sensing LuxR-type transcriptional regulator, EasR in Enterobacter asburiae strain L1
Source: PeerJ. 2020 Oct 21;8:e10068. doi: 10.7717/peerj.10068 (PMC7585371; doi:10.7717/peerj.10068)
Supplement: Supplemental Information 2 — Bacterial strains and plasmids used in this study and the functional motifs in EasR predicted by MOTIF software. [file peerj-08-10068-s002.docx]

**Supplementary Table S1**

**Table S1.** Bacterial strains and plasmids used in this study

| **Strain/plasmid** | **Genotype/description** | **Source/reference** |
| --- | --- | --- |
| *E. asburiae* strain L1 | Lettuce isolate. QS strain with the formation of purple violacein pigment in the presence of short chain exogenous AHL molecules. | (Lau et al., 2013) |
| ***E. coli*** |  |  |
| DH5α  TOP10 | Host without presence of plasmid that yield high quality and concentration of inserted plasmid. dl*ac*Z∆M15 ∆(*lacZYA-argF*)U169 *rec*A1 *end*A1 hsdR17(*rK^-^mK^+^*) *supE*44 *thi-1 gyrA96 relA1*.  Host without presence of plasmid that yield high quality and concentration of inserted plasmid. F- *mcrA* ∆(*mrr-hsd*RMS-*mcr*BC) ф80*lac*Z∆M15 ∆l*ac*X74 *rec*A1 *ara*D139 ∆(*ara*A-leu)7697 *gal*U *gal*K *rps*L (Str^R^) *end*A1 *nup*G | Invitrogen, USA  Invitrogen, USA |
| **Plasmids** |  |  |
| pGEM^®^-T | Toxin-antitoxin (TA) cloning vector, Amp^R^. | Promega, USA |
| pGEM^®^-T-*easR* | pGEMT containing 693 bp of *easR* with NdeI-BglII sites | This study |
| pMULTIAHLPROM | pMP220-derived Broad-host-range plasmid containing 8-*luxI* type promoters *(luxI*, *cviI*, *ahlI*, *rhlI*, *cepI*, *phzI*, *traI and ppuI)* fused to a promoterless *lacZ* gene, Tet^R^. | (Steindler et al., 2008) |
| pLNBAD  pLNBAD-*easR*  TOP10-pMULTI-pLNBAD-*easR* | Multiple cloning site; contains PBAD promoter; Cm^R^.  pLNBAD containing 693 bp of *easR* with NdeI-BglII sites; Cm^R^  pMULTIAHLPROM containing pLNBAD cloned with 693 bp of *easR*; Tet^R^ and Cm^R^ | (Lemonnier et al., 2003)  This study  This study |

^a^Amp^R^, Cm^R^ and Tet^R^ indicate resistance to ampicillin, chloramphenicol, and tetracycline, respectively

**Supplementary Table S2**

**Table S2.** Functional motifs in EasR predicted by MOTIF software

| Pfam | Motif | Position/  (Independent E-value)* | Recognition sequence |
| --- | --- | --- | --- |
| Autoind_bind | Autoinducer binding  domain | 19…152/ (6×10^-18^) | LDAFFEDFKGIVFAYAIMNKKDPSQMRIINNSPEWFDIYLDRKYQFIDPVIIRALRCVEDFFWESDVILSDGYNLTRIFNESVQYDIYQGQTFPLHDYLNNLVVLSVISPKHSGIDIEKYRPQFLSFLVQLHQK |
| GerE | Bacterial regulatory  proteins, *luxR* family | 168…220/ (1.1×10^-20^) | LSPRERQILKWVSAGKTYAEISVILSIAERTVKFHMGNVMKKLGVNNARHAIK |
| Sigma-70_r4_2 | Sigma-70, region 4 | 168…210/ (5.4×10^-6^) | LSPRERQILKWVSAGKTYAEISVILSIAERTVKFHMGNVMKKL |
| Sigma-70_r4 | Sigma-70, region 4 | 168…210/ (0.00033) | LSPRERQILKWVSAGKTYAEISVILSIAERTVKFHMGNVMKKL |
| HTH_23 | Homeodomain-like  domain | 174…199/ (0.09) | QILKWVSAGKTYAEISVILSIAERTV |
| HTH_38 | Helix-turn-helix  domain | 168…199/ (0.11) | LSPRERQILKWVSAGKTYAEISVILSIAERTV |

*E-value represent the probability that a sequence could arise randomly by chance, values below 0.01 could be

of random appearance.
